# Cardiovascular phenotypes of children and adolescents with Turner syndrome from a single-center cohort study

**DOI:** 10.1186/s13023-025-04137-w

**Published:** 2025-12-29

**Authors:** Feihan Hu, Yirou Wang, Yao Chen, Xueqiong Xu, Yu Ding, Lingwen Ying, Qianwen Zhang, Libo Wang, Yuqi Zhang, Lijun Chen, Xiumin Wang

**Affiliations:** 1 Department of Endocrinology, Metabolism and Genetics, Shanghai Children’s Medical Center, Shanghai Jiao Tong University School of Medicine, Shanghai, 200127 China; 2 Department of Pediatric Cardiology, Shanghai Children’s Medical Center, Shanghai Jiao Tong University School of Medicine, Shanghai, 200127 China

**Keywords:** Turner syndrome, Aortic coarctation, Karyotype, Partial anomalous pulmonary venous return, Persistent left superior vena cava

## Abstract

**Background:**

Approximately half of the patients with Turner syndrome (TS) have congenital or acquired cardiovascular diseases. The objectives of this study were to improve the early diagnosis of TS and to predict the risk of severe cardiovascular diseases in patients with TS by analyzing the main features of cardiovascular diseases in these patients.

**Methods:**

This study included 107 patients with TS who underwent echocardiography and were admitted to the Shanghai Children’s Medical Center between November 2019 and November 2024. In this study, the height, weight, age, karyotype, cardiac imaging data, and electrocardiograms of the patients were collected. The main features of the cardiovascular diseases in patients with TS were assessed, and the correlations between the height, body mass index (BMI), karyotype, and cardiovascular diseases were analyzed.

**Results:**

Overall, 107 patients with TS were included, with an average age of 9.68 ± 4.23 years at diagnosis. Bicuspid aortic valve (BAV), aortic coarctation (CoA), and persistent left superior vena cava (PLSVC) were the most prevalent cardiovascular diseases, with prevalence rates of 11.2% (12/107), 8.4% (9/107), and 9.3% (10/107), respectively. The partial anomalous pulmonary venous return (PAPVC) was of the ‘supra cardiac type’ in all cases. Patients with CoA had lower BMI-for-age Z-score (BMIAZ) (-0.81 ± 1.17 vs. 0.61 ± 0.99; *P* = 0.003) than those without CoA. The best cutoff for predicting CoA was calculated using a sensitivity of 0.857 and specificity of 0.697 with a BMIAZ of 0.115 and an area under the curve of 0.825 (*P* = 0.004). Moreover, CoA (63.3%, 7/11) was the leading cause for surgical and interventional procedures in children and adolescents with TS. The 45,X karyotype was found to be associated with congenital heart disease (CHD) (47.4% vs. 24.6%; *P* = 0.016).

**Conclusions:**

Girls with BAV or CoA should undergo karyotyping to facilitate the early diagnosis of TS. CoA is the leading cause for surgical and interventional procedures in children and adolescents with TS. The BMIAZ is a reliable predictor of CoA. Cardiovascular magnetic resonance (CMR) or computed tomography (CT) should be performed at the time of diagnosis for girls with TS, especially those with a 45,X karyotype, BMIAZ < 0.115, or CHD detected by echocardiography.

## Introduction

Turner syndrome (TS) is a genetic disorder caused by the complete or partial absence of a second sex chromosome that affects approximately 25–50 per 100,000 newborn female infants [1–4]. The most common karyotype for TS is 45,X (40%–50%), followed by 45,X/46,XX (15%–25%), 45,X/47,XXX, and X chromosomal structural variants [1, 2]. Clinically, patients with TS present with short statures, primary ovarian insufficiency, skeletal anomalies, neurocognitive issues, and cardiovascular diseases. Approximately half of patients with TS have congenital or acquired cardiovascular diseases, mainly bicuspid aortic valve (BAV), aortic coarctation (CoA), aortic dilation (AD), and partial anomalous pulmonary venous return (PAPVC) [5]. Congenital heart disease (CHD) is the most common cause of death in patients with TS. The karyotype affects the severity of the phenotypic features, and the phenotypes of mosaicism are milder [6]. The cardiovascular disease prevalence is correlated with the karyotype, and 45,X is associated with BAV, PAPVC, persistent left superior vena cava (PLSVC), and CoA [7].

An initial cardiovascular magnetic resonance (CMR) scan is recommended for every patient with TS but can be delayed until it can be performed without general anesthesia in infants and children [1]. However, in clinical practice in China, some patients have poor compliance with CMR because of the high cost and need for anesthesia. Ensuring that all patients undergo CMR is challenging. Understanding the clinical characteristics of patients with cardiovascular diseases may further highlights the necessity of CMR examinations and facilitate improvements in patient compliance. This study aims to improve the early diagnosis of TS and predict the risk of cardiovascular disease requiring surgical and interventional procedures in patients with TS. The data in this study were obtained from the Shanghai Children’s Medical Center, which annually performs more than 77,000 echocardiographies for congenital heart disease, more than 1,700 cardiovascular angiographies, and more than 3,700 cardiac surgeries. These high volumes ensure the robustness of the detection rate, scientific evaluation, and standardization of surgical and interventional procedures for cardiovascular diseases.

## Methods

### Study participants

**Fig. 1:**
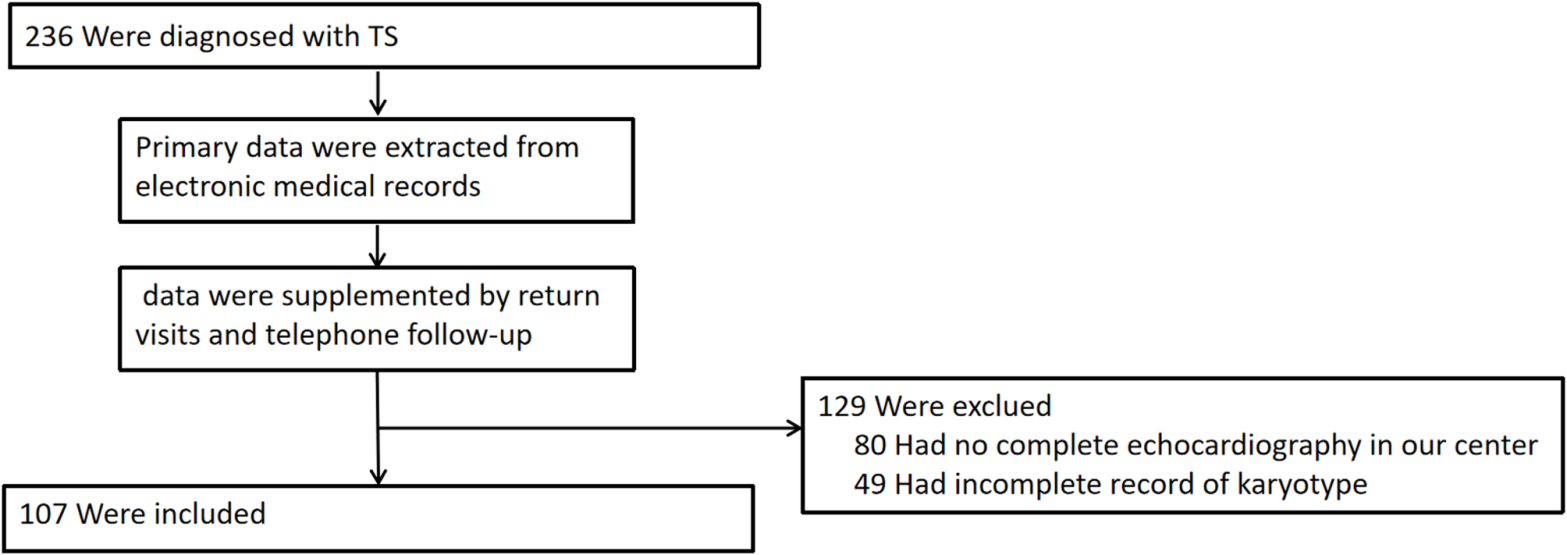
Flowchart of patient selection. TS, Turner syndrome

In this study, patients with TS who underwent echocardiography and were admitted to Shanghai Children’s Medical Center between November 2019 and November 2024 were included. Primary data were extracted from electronic medical records, supplemented by return visits and telephone follow-up. Of the 236 patients with TS who were diagnosed by karyotyping, 129 patients with missing clinical data were excluded. In total, 107 patients underwent echocardiography in our center and had an intact karyotype record were included (Fig. 1). Information, including age at diagnosis of TS, age at diagnosis of CHD, age at the time of cardiac imaging, as well as height and weight at cardiac imaging, chromosome karyotype, and echocardiography, CMR, cardiovascular computed tomography (CT), digital subtraction angiography, cardiovascular angiography, and electrocardiography data, was collected by systematically reviewing cases and examination reports. Imaging utilization was as follows: CMR (1.9%, 2/107), cardiac CTA (13.1%, 14/107), and DSA (2.8%, 3/107). The studies involving human participants were reviewed and approved by The Research Ethics Committee of Shanghai Children’s Medical Center School of Medicine, Shanghai Jiao Tong University (SCMCIRB-K2024208-3). Written informed consent to participate in this study was provided by the participants’ legal guardian/next of kin.

### Cardiovascular evaluation

A Philips iE33 ultrasonic diagnostic apparatus (Philips, Andover, MA, USA), equipped with s5-1 transducer was used to acquire images and loops. These were taken in three-beat capture to optimally visualize the endocardial borders of the heart and vessel at 60 to 100 frames/s at end-expiration. A GE Lightspeed4-slice spiral CT and a GE Signa 1.5T MR were used. CMR was performed with a 2-angle electrocardio-gated spin echo TIW sequence and a 1-angle electrocardio-gated gradient echo movie sequence. Patients were examined using 12-lead combined table electrocardiogram, focused on the QT interval. Hodges formulas were used to correct heart rate and calculate corrected QT interval (QTc) [8, 9]. QTc >450 ms in girls and QTc >460 ms in women were cognized as QTc prolongation [10].

### Statistical analysis

Continuous variables are presented as mean ± standard deviation, and categorical variables are summarized as frequencies and percentages. Independent sample t-tests and one-way analysis of variance were used to compare continuous variables with normal distribution and homogeneity of variance. The chi-square test was used for categorical variables, and in cases of an expected value < 5 a Fischer’s exact test was conducted. The binary logistic regression method was performed for correlation analysis. Receiver operating characteristic (ROC) curves were used to assess the predictive value of the variables for disease. The area under the curve (AUC) was calculated, and a cutoff for each model was estimated by Youden’s J statistic. A p-value of less than 0.05 was considered statistically significant. All statistical analyses were performed using the IBM SPSS Statistics for Windows version 27.0. (IBM Corp., Armonk, NY).

## Results

### Demographic data

For the 107 included patients, the mean age at diagnosis of TS was 9.68 ± 4.23 years, mean height-for-age Z-score (HAZ) was − 1.79 ± 1.30, and mean body-mass-index-for-age Z-score (BMIAZ) was 0.52 ± 1.01 [11].

**Table 1:** Prevalence of CHDs and age at diagnosis of the patients

	Age at diagnosis			Prevalence in this cohort (%)	Prevalence in latest guidelines^1^ (%)	Prevalence of the general population^1^ (%)
	Age at diagnosis with CHD (years)	Age at diagnosis with TS (years)	*P*-value			
CHD	7.42 ± 4.18	8.79 ± 4.12	0.017	32.7	23–50	0.8
Type of CHD						
BAV	6.92 ± 4.00	9.29 ± 3.12	0.031	11.2	14–40	1–2
CoA	5.63 ± 5.22	9.35 ± 3.19	0.033	8.4	4–15	0.34
PAPVC	9.19 ± 1.46	8.34 ± 4.30	0.589	5.6	4–16	0.4–0.7
PLSVC	8.71 ± 2.89	9.19 ± 2.72	0.063	9.3	2–13	0.3–0.5
ARSA	5.50 ± 3.24	5.29 ± 3.62	0.754	3.7	6–8	0.5–2.5

CHD, congenital heart disease; BAV, bicuspid aortic valve; CoA, aortic coarctation; PAPVC, partial anomalous pulmonary venous return; PLSVC, persistent left superior vena cava; ARSA, aberrant right subclavian artery

The results of the prevalence of CHDs in the 107 patients are shown in Table 1. Patients with TS and CHDs were diagnosed with a CHD earlier than they were diagnosed with TS (1.38 ± 3.25 years; *P* = 0.017), particularly pronounced in BAV and CoA. The overall CHD prevalence was 32.7% (35/107). BAV, CoA, and PLSVC had the highest prevalence rates of 11.2% (12/107), 8.4% (9/107), and 9.3% (10/107), respectively. In addition to the CHDs shown in Table 1, other CHDs included patent ductus arteriosus (PDA) (7/107), patent foramen ovale (7/107), atrial septal defect (4/107), ventricular septal defect (1/107), and mesocardia (1/107). Furthermore, the patterns of blood flow in the six patients with PAPVC were as follows: left superior pulmonary vein drainage into the innominate vein (33.3%, 2/6), right superior pulmonary vein drainage into the right superior vena cava (33.3%, 2/6), and left superior pulmonary vein drainage into the left superior vena cava (33.3%, 2/6).

### Aortic coarctation

Nine of all patients had CoA (Table 2). Among them, two patients did not undergo surgical or interventional procedures. One patient had a pressure gradient of 17 mmHg on echocardiography and no clinical symptoms. The other patient had no clinical symptoms, and the assessment of the peak-to-peak pressure gradient during cardiac catheterization was mild. Of the seven patients with CoA who underwent surgical or interventional procedures, six underwent surgical repair. The clinical manifestations in all six patients were ameliorated after surgical repair. Of these six patients, one who experienced severe residual obstruction underwent balloon angioplasty twice at the postoperative follow-up; three patients presented with mild residual obstruction; and no other cardiovascular complications were observed. One patient underwent balloon angioplasty, and the CoA remained at the postoperative follow-up.

**Table 2:** Characteristics of nine patients with coa

Location of CoA	Comorbid Conditions	Surgical or interventional operation	Age of operation (years)	Follow-up time (years)	Preoperative pressure gradient (mmHg)	Preoperative LVEF (%)	Postoperative pressure gradient (mmHg)	Postoperative LVEF (%)
Descending aortic	BAV, PDA, PLSVC, ARSA	-	N/A	N/A	17	73.0	N/A	N/A
Descending aortic	BAV, PDA, AS	Surgical repair	4.8	5.8	56	75.1	17	73.1
Descending aortic	BAV, PDA, PFO, ASD, PAH	Surgical repair	0.4	10.8	64	54.1	31	76.9
Descending aortic	BAV, PAPVC	Balloon angioplasty	N/R	N/R	N/R	N/R	38	64.4
Descending aortic	BAV, PAPVC, AD, PLSVC	Surgical repair	N/R	N/R	N/R	N/R	16	78.8
Descending aortic	-	Surgical repair	0.4	7.8	102	77.4	13	69.7
Descending aortic	PAPVC, PLSVC	-	N/A	N/A	24	65.3	N/A	N/A
Aortic isthmus	MS	Surgical repair	14.2	0.1	N/A	70.8	22	76.0
Descending aortic	PDA	Surgical repair	0.4	9.3	59	72.6	21	63.9

CoA, aortic coarctation; BAV, bicuspid aortic valve; PDA, patent ductus arteriosus; PLSVC, persistent left superior vena cava; ARSA, aberrant right subclavian artery; AS, aortic valve stenosis; PFO, patent foramen ovale; ASD, atrial septal defect; PAH, pulmonary arterial hypertension; PAPVC, partial anomalous pulmonary venous return; AD, aortic dilation; MS, mitral stenosis; N/R, not reported; N/A, not applicable

In addition, the CoA in patients with TS was mostly in the descending aorta. The prevalence of CoA was higher in patients with concomitant CHDs, including BAV (41.7% vs. 4.2%; *P* < 0.001), PDA (57.1% vs. 5.0%; *P* < 0.001), PAPVC (50.0% vs. 5.9%; *P* = 0.007), and PLSVC (30.0% vs. 6.2%; *P* = 0.037) (Table 3).

**Table 3:** Correlation between coa and other CHDs

CHD	Proportion of CoA, % (*n*/*N*)		*P*-value
	Proportion in patients with specific CHD	Proportion in patients without specific CHD	
BAV	41.7 (5/12)	4.2 (4/95)	< 0.001
PAPVC	50.0 (3/6)	5.9 (6/101)	0.007
PLSVC	30.0 (3/10)	6.2 (6/97)	0.037
ARSA	25.0 (1/4)	7.8 (8/103)	0.300
PDA	57.1 (4/7)	5.0 (5/100)	< 0.001

CHD, congenital heart disease; CoA, aortic coarctation; BAV, bicuspid aortic valve; PAPVC, partial anomalous pulmonary venous return; PLSVC, persistent left superior vena cava; ARSA, aberrant right subclavian artery; PDA, patent ductus arteriosus

**Fig. 2:**
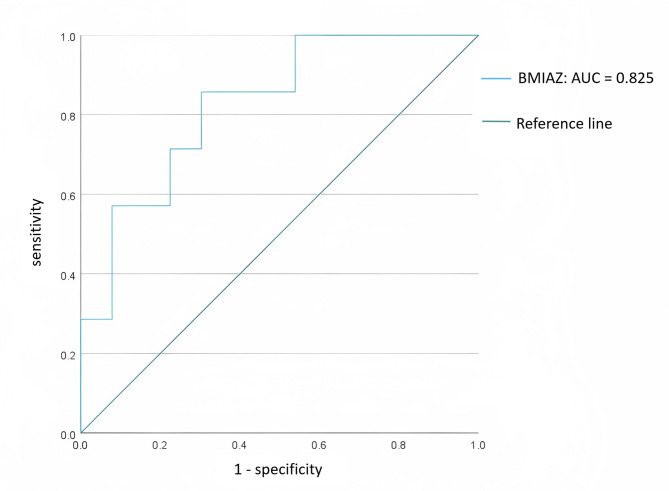
ROC curves depicting prediction of CoA by BMIAZ. BMIAZ, body-mass-index-for-age Z-score; AUC, area under the curve; ROC, receiver operating characteristic; CoA, aortic coarctation

Patients with CoA before surgical or interventional procedures had lower BMIAZ (-0.81 ± 1.17 vs. 0.61 ± 0.99; *P* = 0.003) (Table 4). The AUC using BMIAZ as a predictor of CoA was 0.825 (*P* = 0.004) (Fig. 2). The best cutoff for predicting CoA was calculated as follows: BMIAZ, 0.115; sensitivity, 0.857; and specificity, 0.697.

**Table 4:** Comparative analysis of the preoperative height and BMI of patients with or without coa

	Mean ± standard deviation		OR	95% CI of OR		*P*-value
	With CoA	Without CoA		Lower	Upper	
HAZ	-1.74 ± 1.34	-1.77 ± 1.32	1.018	0.574	1.808	0.951
BMIAZ	-0.81 ± 1.17	0.61 ± 0.99	0.247	0.099	0.617	0.003

CoA, aortic coarctation; BMI, body mass index; height-for-age Z-score, HAZ; BMI-for-age Z-score, BMIAZ

Overall, 8 (7.5%) of the 107 patients underwent surgical procedures, and 3 (2.8%) of patients underwent interventional procedures. The characteristics of the other four patients without CoA who underwent surgical procedures are shown in Table 5. None of the four patients experienced postoperative complications during the follow-up period. CoA (63.3%, 7/11) was the leading cause of the surgical and interventional procedure. CoA was a significant risk factor for surgical and interventional procedures (77.8% [7/9] vs. 4.1% [4/98]; *P* < 0.001).

**Table 5:** Characteristics of the four surgical patients without coa

Lesions	Surgical or interventional operation	Age of operation (years)	Follow-up time (years)	Preoperative LVEF (%)	Postoperative LVEF (%)
PDA	Surgery	2.2	4.3	65.0	69.5
ASD	Intervention	10.3	0.2	65.1	68.8
PDA	Intervention	9.2	4.0	58.6	61.8
Mesocardia, BAV, AS, PLSVC	Surgery	5.0	7.0	73.9	68.1

PDA, patent ductus arteriosus; ASD, atrial septal defect; BAV, bicuspid aortic valve; AS, aortic valve stenosis; PLSVC, persistent left superior vena cava

### Karyotypes

The 45,X karyotype was associated with CHD (47.4% vs. 24.6%; *P* = 0.016). Patients with a 45,X karyotype had a higher prevalence of BAV (23.7% vs. 4.3%; *P* = 0.007), PAPVC (15.8% vs. 0.0%; *P* = 0.003), and aberrant right subclavian artery (ARSA) (10.5% vs. 0.0%; *P* = 0.027). The proportion of CoA was higher in patients with a 45,X karyotype; however, the difference was not significant (15.8% vs. 4.3%; *P* = 0.094) (Table 6).

**Table 6:** Correlation between CHD and karyotypes

Lesion	Total	Karyotypes		
		45,X *N* = 38	Other karyotypes *N* = 69	*P*-value
BAV, n (%)	12 (11.2)	9 (23.7)	3 (4.3)	0.007
CoA, n (%)	9 (8.4)	6 (15.8)	3 (4.3)	0.094
PAPVC, n (%)	6 (5.6)	6 (15.8)	0 (0.0)	0.003
PLSVC, n (%)	10 (9.3)	5 (13.2)	5 (7.2)	0.510
ARSA, n (%)	4 (3.7)	4 (10.5)	0 (0.0)	0.027
PDA, n (%)	7 (6.5)	3 (7.9)	4 (5.8)	0.991
PFO, n (%)	7 (6.5)	2 (5.3)	5 (7.2)	1.000
ASD, n (%)	4 (3.7)	0 (0.0)	4 (5.8)	0.327
VSD, n (%)	1 (0.9)	1 (2.6)	0 (0.0)	0.355

CHD, congenital heart disease; BAV, bicuspid aortic valve; CoA, aortic coarctation; PAPVC, partial anomalous pulmonary venous return; PLSVC, persistent left superior vena cava; ARSA, aberrant right subclavian artery; PDA, patent ductus arteriosus; PFO, patent foramen ovale; ASD, atrial septal defect; VSD, ventricular septal defect

### Other pertinent aspects

Overall, there was slight left heart enlargement in 8 (7.5%) of 107 patients and slight left ventricular wall thickening in 5 (4.7%), none of those paitents had cardiac dysfunction. The mean left ventricular ejection fraction (LVEF) of all patients was 69.36 ± 5.00%. None exhibited impaired systolic function.

Of the 107 patients, 78 (72.9%) underwent electrocardiography. Common arrhythmias in patients with TS included sinus arrhythmias (14.1%, 11/78), sinus tachycardia (9.0%, 7/78), T-wave abnormalities (10.3%, 8/78), and ST-wave abnormalities (9.0%, 7/78) (Table 7). The mean QTc of all patients was 419.29 ± 18.98 ms, and 6 of 78 patients (7.7%) had QTc prolongation. Other arrhythmias included A-V junctional premature beats (3.8%, 3/78) and ventricular preexcitation (2.6%, 2/78). There was no significant correlation between the arrhythmias and karyotypes.

**Table 7:** Prevalence of arrhythmia in 78 patients

Arrhythmia	Total *N* = 78	Karyotypes		
		45,X *N* = 25	Other karyotypes *N* = 53	*P*-value
QTc prolongation, n (%)	6 (7.7)	3 (11.5)	3 (5.8)	0.652
T-wave abnormalities, n (%)	8 (10.3)	3 (11.5)	5 (9.6)	1.000
ST-wave abnormalities, n (%)	7 (9.0)	3 (12.0)	4 (7.7)	0.889
Sinus arrhythmia, n (%)	11 (14.1)	2 (7.7)	9 (17.3)	0.421
Sinus tachycardia, n (%)	7 (9.0)	4 (15.4)	3 (5.8)	0.327

## Discussion

TS is caused by the complete or partial absence of a second X chromosome. It is estimated that the 45,X karyotype accounts for 40%–50% of patients with TS [1, 2] and for 39.2% of patients with TS in the Chinese population [12]. The 45,X karyotype accounted for 32.1% of the patients with TS in this study, which may be because more patients with mosaicism were diagnosed at our center, which had adopted a more active karyotyping of girls with short statures. Patients with TS demonstrate significantly reduced height and increased BMI compared to the general female population [2, 13], which is consistent with the findings of our study.

The prevalence of CHDs in patients with TS ranges from 23% to 50%, and left-sided obstructive lesions such as BAV and CoA are the most common, which is consistent with the findings of our study. Our study indicates that CHDs were typically diagnosed before TS in affected patients. Thus, karyotyping of patients with CHDs may facilitate the early diagnosis of TS, particularly in those with BAV or CoA. The necessity of karyotyping in females with BAV or CoA has been recognized [1, 14, 15]. Moreover, PAPVC, PLSVC, and ARSA are prevalent in patients with TS [1, 16]. In our study, PLSVC, which is generally considered a normal variant, was significantly more prevalent (9.3%, 10/107) than in the general population (0.3%–0.5%). Therefore, karyotyping in females with PLSVC would be valuable. In addition, ‘supra cardiac type’ PAPVC(i.e. pulmonary veins draining into the superior caval vein or innominate vein) is highly prevalent in patients with TS and uncommon in the general population [16]. In our study, all patients with PAPVC presented with the ‘supra cardiac type’. Thus, ‘supra cardiac type’ PAPVC is suggestive of TS.

### Aortic coarctation

CoA is a common CHD in patients with TS [5, 17]. Previous studies have shown that the CoA correlates with other CHDs, including BAV, Shone’s complex, and PDA [17, 18]. In isolated CoA, ingrowth of the ductus tissue results in constriction [19]. In CoA associated with other CHDs, the main etiology is inadequate growth, as upstream obstructions lead to decreased fetal flow through the aorta [17]. In our study, CoA in patients with TS was associated with other CHDs, including BAV, PDA, PAPVC, and PLSVC. In addition, severe CoA in infants and children can lead to inadequate systemic circulatory perfusion, causing growth retardation [17]. Patients CoA showed a significantly lower BMI than those without CoA. Therefore, in patients with TS and a BMIAZ < 0.115, especially in those with multiple cardiovascular diseases, attention should be paid to the possible presence of CoA. CoA which is very distally located or which has mild nature may not be seen by echocardiography [20], and performing CMR or CT in these patients is especially important.

CoA is the leading cause of surgical and interventional procedures in patients with TS. Untreated CoA can lead to serious complications such as hypertension, heart failure, and aortic dissection [15, 17]. Surgery is necessary for most patients with TS and CoA. Patients with CoA, associated with hypertension in cases of an upper-to-lower-extremity blood pressure gradient or a mean CoA gradient ≥ 20 mmHg on echocardiography (or ≥ 10 mmHg with left ventricular dysfunction, aortic insufficiency, or collaterals) are recommended to proceed with surgical or interventional procedures [21]. For children with symptoms of chronic cardiac insufficiency, such as respiratory effort, feeding difficulties, and lagging growth, surgical or interventional procedures should be performed after pharmacological therapy [17]. Intravascular stent placement, surgical repair, and balloon angioplasty are performed in patients with CoA, and surgical and interventional procedures should be carefully chosen. Intravascular stent placement is the preferred treatment for CoA in adults and late adolescents [17]. Balloon angioplasty has short-term effectiveness but is associated with higher rates of recoarctation and aneurysm formation [22, 23], especially in patients with TS [24, 25]. Surgical repair may be the most effective treatment option for children and adolescents with TS.

### Other pertinent aspects

Cardiac function in patients with TS has rarely been reported. In our study, the overall cardiac function of the patients was generally normal. A few patients with severe cardiovascular diseases had a slight decrease in cardiac function. Surgical and interventional procedures for severe cardiovascular diseases improve the prognosis of cardiac function.

Karyotypes can affect the severity of phenotypic features. Mosaicism phenotypes are milder [6]. The morbidity and mortality rates of patients with TS vary according to karyotype. The 45,X karyotype increases the rate of death by nearly 5-fold compared to that in the general population, and the other karyotypes increase the rate of death by 2- to 4-fold [26]. The prevalence of cardiovascular diseases has been correlated with the karyotype, and 45,X is associated with BAV, PAPVC, PLSVC, and CoA [7]. In our study, the 45,X karyotype was associated with BAV, PAPVC, and ARSA. The prevalence of CoA was higher in patients with the 45,X karyotype; however, the difference was not statistically significant.

Patients with TS are more prone to arrhythmias [27], including sinus tachycardia, T-wave abnormalities, and ST-wave abnormalities, than the general population, and most have no clear clinical significance [1, 2, 27]. The Hodges formula is recommended for heart rate corrections when calculating the QTc [1]. QTc prolongation is defined as a QTc >450 ms in girls and >460 ms in women [8, 9]. Patients with TS are recommended to consult a cardiologist for QTc prolongations >480 ms that are detected on at least two consecutive electrocardiograms [1]. The QTc is not significantly different between patients with TS and the general female population, which is approximately 5% [10]. In our study, 6 of 78 patients had QTc prolongation (7.7%), which was not significantly different from that in the general female population.

### Limitations

Our study had some limitations. The data in this study were obtained from the National Children’s Medical Center, which has the best echocardiography technology, the most experienced echocardiography specialists, and the strictest quality control of echocardiographic reports in China, which guarantees the optimal detection rate of cardiovascular diseases. Patients have low CMR compliance because of the high cost and need for anesthesia. Our center uses echocardiography as a mandatory examination for patients with TS at the time of diagnosis. Patients with severe cardiovascular diseases and unclear echocardiography findings undwent CMR, CT, and cardiac catheterization, according to the recommendations of the cardiologists. This may have resulted in missed diagnoses of mild CHDs. In addition, our study was a single-center study with a limited number of patients with TS. Further exploration will include a multicenter study.

## Conclusion

We studied the cardiovascular status of 107 children and adolescents with TS at a single center in China. ‘Supra cardiac type’ PAPVC and PLSVC, in addition to BAV and CoA, are characteristic of patients with TS [1]. Therefore, girls with BAV, CoA, ’supra cardiac type’ PAPVC or PLSVC should undergo karyotyping. It is particularly critical for those with BAV or CoA to facilitate the early diagnosis of TS. Moreover, CoA is the leading cause for surgical and interventional procedures in patients with TS. Patients CoA have a lower BMI, and the BMIAZ may be a reliable predictor of CoA. Completing CMR or CT at the time of diagnosis is urgently recommended, especially in patients with a 45,X karyotype, BMIAZ < 0.115, or CHDs detected by echocardiography.

## Data Availability

The data that support the findings of this study are available from the corresponding authors upon reasonable request.

## Abbreviations

AD: Aortic dilation
ARSA: Aberrant right subclavian artery
AS: Aortic valve stenosis
ASD: Atrial septal defect
AUC: Area under the curve
BAV: Bicuspid aortic valve
BMI: Body mass index
BMIAZ: BMI-for-age Z-score
CHD: Congenital heart disease
CMR: Cardiovascular magnetic resonance
CoA: Aortic coarctation
CT: Computed tomography
HAZ: Height-for-age Z-score
LVEF: Left ventricular ejection fraction
MS: Mitral stenosis
PAH: Pulmonary arterial hypertension
PAPVC: Partial anomalous pulmonary venous return
PDA: Patent ductus arteriosus
PFO: Patent foramen ovale
PLSVC: Persistent left superior vena cava
QTc: Corrected QT interval
ROC: Receiver operating characteristic
TS: Turner syndrome
VSD: Ventricular septal defect
